# 

**Published:** 1889-09-21

**Authors:** 


### NOTICE TO CORRESPONDENTS.

All MS., Letters, Books for Review, and other matters intended for the Editor, should be addressed The Editor, The Lodge, Porchester Square, London, W.

### Notice to Advertisers and Subscribers.

All Advertisements, Orders for Copies of The Hospital, and Business Communications should be addressed to The Publisher, *not to the Editor*, The Hospital, 139-140, Salisbury-court, Fleet-street, E.C.

Vol. V., now ready, 4s., post free. Cases for binding, may be had of the Publisher, at 1s. 6d. each.

### To Residents, Secretaries, and Matrons.

The Editor will be glad to have the Regulations and each Report as issued by Asylums, Hospitals, Convalescent and Nursing Institutions, as well as those of other Charities, and an early copy in duplicate of all Appeals and Festival Notices, etc., addressed to *The Editor, The Lodge, Porchester-square, London, W.* Any superintendent, secretary, matron, or other chief official who regularly sends such information may be registered, on application to the Publisher, to receive free of cost a cover or binding The Hospital in half-yearly volumes.

### Amusements and Relaxation.

### SPECIAL NOTICE.

#### ACROSTIC COMPETITION.

The Prize Editor having been requested by several correspondents to continue the *Original* and *Set* Acrostic Competitions will be glad to receive the names of all intending competitors on or before October 3rd.

### Second Quarterly Word Competition Commenced July 6th, ends September 28th.

Three Prizes of 15s., 10s., 5s., will be given for the largest number of words derived from the words set for dissection.

Proper names, abbreviations, foreign words, words of less than four letters, and repetitions are barred; plurals, and past and present participles of verbs, are allowed. Nuttall's *Standard* dictionary only to be used.

N.B.—Word dissections must be sent in WEEKLY, arranged alphabetically, with correct total affixed.

The word for dissection for this, the *Twelfth* week of the quarter, being "B A L M O R A L."

#### Results of Tenth Week.

| Names. | Sept. 12th. | Totals. | Names. | Sept. 12th. | Totals. |
|---|---|---|---|---|---|
| Nurse Duty | 59 | 555 | Isabel | 53 | 522 |
| Jumps | — | 30 | F. C. J. | – | 305 |
| N'importe Qui | 51 | 505 | Percy Verance | — | 18 |
| Tinie | — | 186 | Gleniffer | — | 175 |
| Amicus | — | 430 | Gypsye | 53 | 490 |
| Patience | 59 | 561 | Ladyr | — | 127 |
| Broxbourne | — | 398 | A. B. | — | 170 |
| Speedwell | 50 | 504 | Essie | — | 393 |
| Lightowlers | — | 356 | Cowboy Bill | — | 179 |
| Lauriston | — | 345 | M. L. A. | — | 117 |
| Sister | 44 | 521 | W. G. R. | 40 | 428 |
| Rattler | — | 28 | Little Tuppy | — | 110 |
| Paignton | — | 334 | Embryo | — | 56 |
| Fassifern | — | 141 | Jenny Wren | 50 | 437 |
| W. R. E. | 58 | 541 | Pearl | — | 132 |
| Buxton | — | 167 | Condorcet | 30 | 450 |
| Nux Vom | — | 263 | R. W. | — | 43 |
| Esperance | 56 | 551 | Electrician | 58 | 318 |
| Spes | — | 24 | Nurse Amie | — | 89 |
| Secnarf | — | 229 | Sunshine | 47 | 223 |
| M. W. | 55 | 555 | Juvenis | — | 23 |
| Rover | — | 226 | Qu'appelle | 58 | 491 |
| Leirion | — | 195 | K. Jarman | — | 43 |
| Matron | — | 174 | Reveal | — | 196 |

#### Notice to Correspondents.

N.B.—All letters referring to this page which do not arrive at 139 and 140, Salisbury-court, London, E.C., by the *first post on Thursdays*, and are not addressed PRIZE EDITOR, will in future be disqualified and disregarded.

### Scraps and Gleanings.

A probable death from cocaine occurred lately at Liverpool.

Dr. Anuschat advocates warm baths in typhoid fever instead of cold.

Hospital Saturday at Ilfracombe resulted in a collection of nearly £200.

The British Pharmaceutical Conference will be held this year at Newcastle.

Dr. Bennett and Dr. Havard are among the New Pembrokeshir magistrates.

The Marchioness of Dufferin's "Indian Journal" will be published immediately.

A quarterly board of governors of St. Mary's Hospital was held on September 13.

Ada Heather-Bigg writes to the *Times* in favour of women inspectors of factories.

There are now about 400 cottage hospitals in England, furnishing nearly 4,000 beds.

An interesting article, entitled "A Doctor's Outing," appears in this month's *Belgravia*.

Mr. Archdale Lloyd Sharpin has been elected house surgeon of Salisbury Infirmary.

Dr. C. H. West has been appointed house surgeon to the North Cambridgeshire Hospital.

The Great Northern Hospital has received £50 from a police and tradesmen's cricket match.

Over 3,000 persons joined in the friendly societies hospital parade at Teignmouth on August 1st.

Mr. O'Grady, of Mercer's Hospital, has been presented by his friends with a brougham and horse.

Anti-Vivisectionists must be proud and contented now the *Family Herald* has espoused their cause.

Typhoid is rather prevalent in Belfast, owing to the rains of August following on the drought of July.

Vermont Microscopical Society offers a prize of 250 dollars to the first discoverer of a new disease germ.

A demonstration of a large character was held at Morley on August 1st, in aid of Leeds General Infirmary.

The Children's Medical and Surgical Home, Victoria Hospital, Chelsea, will be opened on October 1st.

Mr. Macartney, M.P., has been calling attention to the increased use of ether in Ireland as an intoxicant.

Dr. Billroth, of Vienna, says many wounds are aggravated by the uncalled-for application of carbolic acid.

The *Nineteenth Century* contains an article by Dr. Behrend on "Diseases Caught from Butcher's Meat."

The Viceroy of India offers for competition amongst female medical students, five silver and two bronze medals.

Dr. Mary Jane Hall has just published a most useful and interesting little volume, entitled "Essays for Women."

Forfarshire sends over 100 patients annually to the Edinburgh Infirmary and only contributes £2 to the institution.

Dr. Clifford Allbutt will deliver the introductory address at St. George's Hospital Medical School on October 1st.

The men working at the Manchester Ship Canal are subject to a special sore throat, becoming known now as "canal throat."

Hangnail should really be written angnail, from the Saxon, meaning pain nail; but this is a case in which an "h" has been inserted.

Medical officers of health, it is proposed, should receive special training in their duties, and be obliged to pass a special examination.

Sir Morell Mackenzie's daughters may be seen daily on the Thames indulging in the healthy pursuits of punting and sculling.

Dr. Jablonski says that children suffering, or suspected of suffering, from tuberculosis should not be permitted to attend public schools.

Dr. Jessop's address on "The Treatment of Cancer," read before the British Medical Association, is a clever *resume* of modern treatment.

The Shoreditch Guardians have opened cottage homes at Hornchurch for their pauper children. A hospital and a swimming-bath are amongst the buildings.

Baudelocque, a celebrated French physician, asserted long ago that the repeated breathing of the same atmosphere is a primary and efficient cause of scrofula.

A nurse at Belvedere Fever Hospital while walking along a passage suddenly sank through the ground. The hospital had been built over an old coal-pit shaft.

Deputy Surgeon-General W. R. Rice is appointed Sanitary Commissioner and Surgeon-General to the Indian Government, on the resignation of Sir B. Simpson.

The Isleworth friendly societies' church parade, in aid of the Richmond Hospital, will be held on October 6. The Vicar of Isleworth will preach in the afternoon.

Miss Julia Cock, of Shrewsbury, and Miss Jane Harriet Walker, of Dewsbury, have been admitted licentiates of the Royal Colleges of Physicians and of Surgeons of Edinburgh and Glasgow.

The parcel post to India seems to have struck a native resident in London as a fitting vehicle for the transmission of his brother's ashes, after cremation, for dispersal in the sacred Ganges.

Dr. Greville Macdonald, physician to the Throat Hospital, Golden-square, describes "an aggravated form of chronic congestion of the laryngeal mucous membrane" as attributable to over-work and to straining of the voice by female teachers in London Board schools.

In the small leper-farm near Nikosia, Cyprus, are now isolated some 50 lepers, who are, all but one or two Turks, Greek Christians—which fact it is that lends weight to the popular Turkish belief that the lowest Greeks contract leprosy from eating badly-cured pork, which the heat of the climate reduces to a decayed condition.

